# Normal pressure hydrocephalus secondary to Lyme disease, a case report and review of seven reported cases

**DOI:** 10.1186/s12883-020-01917-8

**Published:** 2020-09-16

**Authors:** Louise Nørreslet Gimsing, Anne-Mette Hejl

**Affiliations:** 1 Department of Specialized Neurorehabilitation, Hvidovre Hospital, Kettegårds Allé 30, 2650 Hvidovre, Denmark; 2 Department of Neurology, Copenhagen University Hospital Bispebjerg, Bispebjerg Bakke, 2400 Copenhagen, Denmark

**Keywords:** Secondary normal pressure hydrocephalus, Chronic Borreliosis, Normal pressure hydrocephalus, Lyme disease

## Abstract

**Background:**

Infection with tick borne Borrelia Burgdorferi (Lyme disease) can without treatment rarely develop into a chronic phase. Secondary Normal Pressure Hydrocephalus (sNPH) based on chronic infection with Borrelia Burgdorferi (Bb) is an even rarer entity, that with the right treatment is potentially curable.

**Case presentation:**

A 67-year-old male with a slow onset of progressive balance problems, also presented unspecified dizziness, urge feeling, neck soreness and discrete cognitive complaints. An MRI scan revealed an enlarged ventricular system compatible with NPH. After further liquor dynamic procedures, cerebrospinal fluid (CSF) was analysed with the surprising results of lymphocytic pleocytosis, and signs of increased antibody production. Microbiology revealed chronic neuroborreliosis and the patient was treated with antibiotics accordingly. At the one-year follow-up no symptoms remained and the ventricular system almost normalized.

**Conclusions:**

We describe the 7th published case of sNPH secondary to chronic Borreliosis in a previous healthy adult. Existing published literature has been reviewed and previous cases showed similarly nearly full clinical recovery. Primary/idiopathic NPH (iNPH) is treated with the surgical intervention of ventriculoperitoneal shunt and can be mistaken for a sNPH. The awareness of rare causes of sNPH like chronic Borreliosis is important as it is easily treated non surgically.

## Background

The chronic phases of infection with the spirochete Borrelia Burgdorferi (Bb), European Lyme disease is characterized by involving several organ systems. Involvement of the nervous system, neuroborreliosis, can develop in untreated individuals usually within 2–6 weeks [1], and typically includes signs of meningeal irritation comprising of nuchal tenderness, fatigue, nausea and the two cardinal symptoms: painful meningoradiculitis and peripheral motor deficits (the clinical part of Bannwarth’s triad [1]).

The rare condition of chronic neuroborreliosis (duration > 6 months) can evolve to a variety of different sub conditions, normal pressure hydrocephalus (NPH) being one of them.

In this article we present a rare adult case of NPH in a prior healthy individual, who turned out to have developed the clinical and radiological syndrome secondary to chronic neuroborreliosis. Full recovery was achieved after antibiotic (AB) treatment.

## Case presentation

**Fig. 1:**
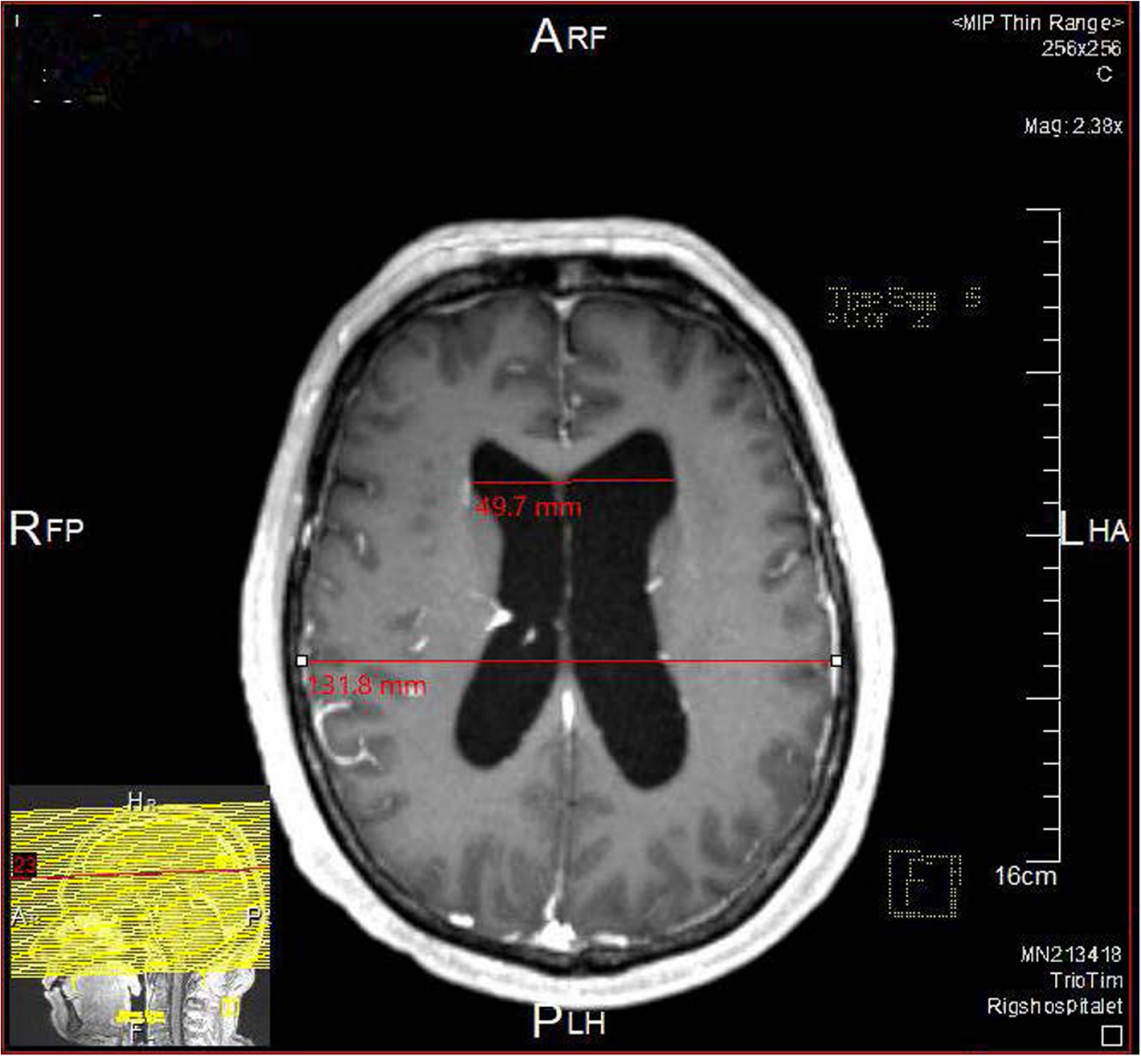
NPH hos case

A 67-year-old male, healthy and with no prior admissions, was seen in autumn of 2009 by his General Practitioner (GP). The patient complained of increasing dysfunctional levels based on several different symptoms, all onset within 3–4 months and progressing slowly. The main complaints included balance problems (no falls reported), diffuse dizziness (not rotatory or nautical) and urinary urge-incontinence. During the same period, he had problems concentrating and with finding words with an increased irritability. He complaint of sore muscles of the arms muscles and around the neck a moderate morning headache (VAS = 5) and an increasing feeling of exhaustion. He had no problems of slow movements, coordination problems, body stiffness, hallucinations, lateralized symptoms nor changes of personality. An MRI scan of the brain without contrast, revealed dilatated lateral and third ventricles, normal forth ventricle and sulci. The radiologist concluded a suspicion of NPH (Fig. 1) based on an Evans index of 0.377 a (DESH signs). The callosal angle was 117 ^o^ and so not significantly decreased. The patient was referred to a neurosurgical clinic, who transferred the patient to a specialized Memory Disorders Clinic for evaluation of possible NPH.

At the first examination here (December 2009) he also reported fragmented sleep during the night. He reported, no exposure of tick, insect bite nor rash.

### Assessment

pt?>On physical examination the neurologist found normal vital signs and no fever. No sign of frontal lope dysfunction. No stiffness to the neck and no skin rash. The neurological examination revealed occasional searching for words, but normal sentence construction and fluency, normal naming of objects. There was no sign of dysarthria. Cranial nerves and muscle strength were normal, but a discrete ataxia in the left arm and leg was found. The patient had no sign of parkinsonism: no tremor, had a normal posture and arm swing in gait. Gait was with slight gait apraxia but with normal speed. He walked with normal speed, broadened, but with normal step length and height. Walking on a straight line caused imbalance. There was a negative test of Romberg. Tonicity of the lower extremities was moderately increased bilaterally, but the deep reflexes, plantar responses and clonus were normal. Primary reflexes of snout and frontal tapping were negative.

On cognitive testing with the Minimal Mental State Examination (MMSE [2]) and Addenbrooke’s Cognitive Examination (ACE [3]) he showed mild cognitive impairment (MMSE = 28/30, ACE = 89/100. Affected animal fluency and s-word fluency.).

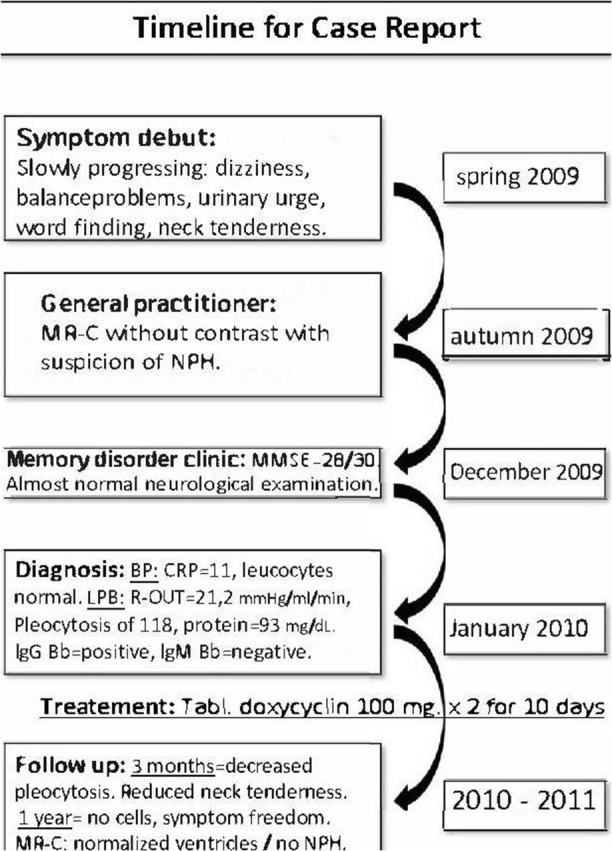



### Diagnosis and management

The initial laboratory test found marginally elevated C reactive Protein (CRP) = 11 and sedimentation reaction = 20. Complete blood cell count, electrolytes, liver enzymes, albumin, creatinine, lactate dehydrogenate, thyroid stimulating hormone, B12, folate and calcium were normal.

In the workup battery of NPH, a lumbar puncture (LBP) was performed with a normal opening pressure (17 mmHg), but a lumbar infusion test (4) with an R-OUT = 21.2 mmHg/ml/min, being diagnostic for NPH (> 16 mmHg/ml/min).

The CSF showed an increased protein count (93 mg/dL, [20–40 mg/dL]), lymphocytic pleocytosis (118 U/mm^3^, 80% lymphocytes) and microscopy with polyform leucocytes but no microorganisms (especially no cryptococcus). Further CSF-analysis showed at first negative oligoclonal bands, positive Bb IgG with a CSF/serum-ratio = 11.7 and no synthesis of Bb IgM.

In lack of enough CSF and with the surprising pleocytosis, an LBP was repeated 4 days after showing additional increases in protein count (101 mg/dL), more pronounced pleocytosis (186 U/mm^3^, 95% lymphocytes) and now positive oligoclonal bands and an IgG CSF/blood-ratio of 12.4.

The CSF was cultured for bacteria and fungus showing neither. Further tests for both HIV (PCR and DNA), cryptococcus (antibodies), HSV and VZV (PCR and antibodies) and mycobacteria and Tuberculosis (PCR) were negative. A flowcytometry showed sign of reactive but not malignant immune response.

Screening for Syphilis and sarcoidosis (S-ACE) tested negative, and a repeated general laboratory test (a month later than the first) showed normalized CRP and again no further abnormality.

The MRI scan of the brain was repeated (4 months after the first), showing unchanged dilated lateral and third ventricles (unchanged EI), persistent periventricular hyperintensity and no post contrast enhancements.

Despite the lack of leptomeningeal enhancement, the positive intrathecal Bb antibody synthesis and the prolonged symptoms gave the diagnosis: chronic neuroborrelioses, and so indicated AB treatment.

As the patient within a week after the first LBP reported slight decrease in symptoms, he was discharged with peroral Doxycyclin, 200 mg. × 2 the first day and hereafter 100 mg. × 2 for 10 days.

### Outcome and follow-up

At 3-month follow-up, a control LBP was performed, with the pleocytosis almost diminished (23 U/mm^3^, 96% lymphocytes), decreased protein count (62 mg/dL) but still showing signs of increased antibody concentrations with elevated unspecified IgG and positivity for oligoclonal bands.

Subjectively the patient reported no more headache, balance problems nor urge. The subjective cognitive problems of concentration and search for words were almost gone, yet occasionally muscle soreness remained.

Another 3 months later, the patient reported neck tenderness as the only symptom. The repeated LBP proved further improvement with only slight pleocytosis (11 U/mm^3^), normalized protein count (58 mg/dL), decreasing unspecified Still present oligoclonal bands.

At the 1- year follow-up (after treatment) the patient was symptom free.

At that point a control LBP showed normal CSF cell count, normal protein count and decreasing immunoglobulins: unspecified IgG = 0.110 g/L, IgG-index = 1.76.

An MRI scan was repeated with now almost normalized ventricle size, normal sulci, no periventricular hyperintensity and still no post contrast enhancement. MMSE was unchanged (29/30) but the patient performed better in ACE (97/100).

The patient was discharged with no further control.

## Discussion and conclusions

NPH was first recognized in 1965, as a syndrome of hydrocephalus with normal CSF opening pressure and with cognitive decline, urinary incontinence and gait disturbance, potentially reversible by neurosurgical procedures [4].

Since 2000 [5] the term secondary NPH in contrast to idiopathic NPH has been used in the literature, and while the definition of iNPH previously was “just” the lack of an explanation [6], later studies [7, 8] have shown, that both the pathophysiology, the type of expansion of the ventricular system [7], the epidemiological characteristics [9] and the prognosis after treatment [9] differs between iNPH and sNPH.

sNPH caused by neuroborreliosis is believed to be triggered in arachnoid granulate in the subarachnoidal spaces when the chronic infection presents itself in the form of infiltration of the spirochete, and when inflammatory cells and proteins disturb the CSF absorption [5]. It falls in the same category as NPH by neurosyphilis [10] and by cryptococcus infection [11]. Infectious sNPH is believed to develop within a time span from 10 days to up to 6 months after the infection [9].

**Fig. 2:**
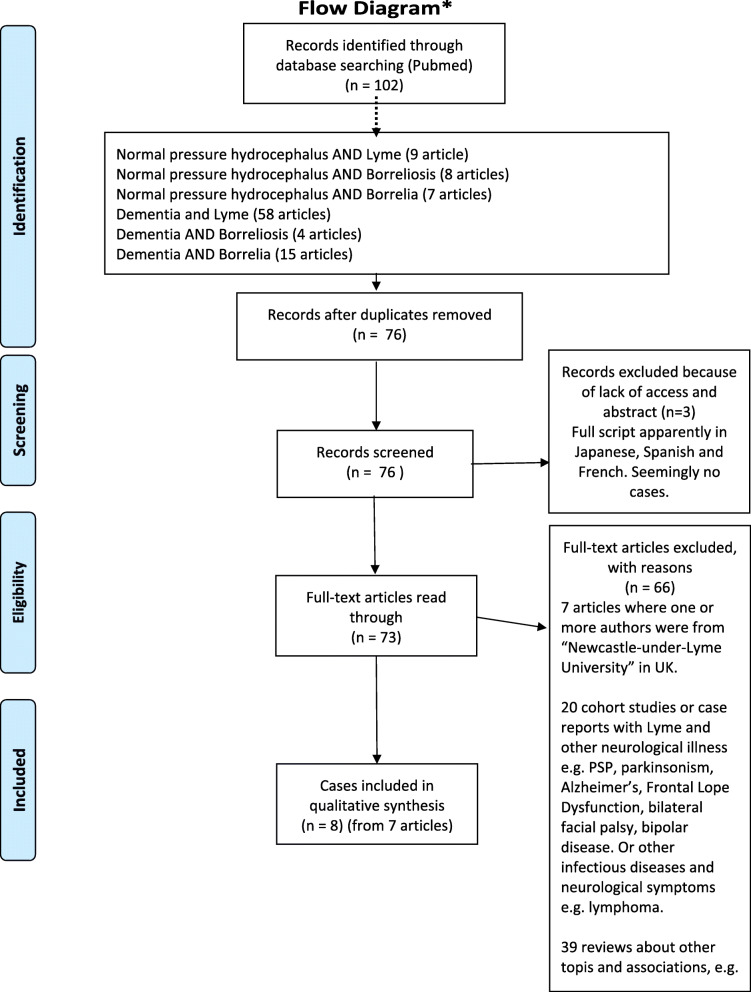
Flow diagram of literature

The first Bb induced sNPH case was described in 1993, published in 1997 [12]. In total there are only eight published cases [12–19] appearing in the Pubmed database, identified by the systematic combination of the search words: Lyme, Borrelia, Borreliosis AND Normal pressure hydrocephalus (also including a non-English articles) (Literature search and selection can be seen in Fig. 2, overview of the articles in Table 1).

**Table 1:** Overview of the characteristics, clinical findings and diagnostics of all published cases to date of sNPH in patients with chronic neuroborreliosis, listed in chronological order

Year of publication	Sex	Age (years)	Duration of symptoms at admission	Symptoms	Clinical findings (incl. Tap-test)	Radiology	Bb IgM CSF	Bb IgG CSF	Bb IgG CSF/ serum-ratio	Pleocytosis (leucocytes, U/mm3)	CSF-protein (mg/dl.)	Differential diagnosis
1996 [12]	Female	76	9 months	General weakness. Progressive gait and memory problems. Debut of urine incontinence.	Delayed broad-based gait with leftward drift. Inability to perform tandem gait or stand on one leg. Reduced attention and memory. Abnormal behaviour. MMSE 20/30. Tap test^a^ (40 ml.) with no effect. No LIT.	MRI = Dilated ventricles not matched by an equal increase of the subarachnoid space. Patches of subependymal signal abnormalit Suggesting NPH.	Positive.	Positive.	12.6	98 (82% lymphocytes).	191	CSF for Treponema pallidum hemagglutination (TPHA) = negative.
1999 [13]	Male	57	> 12 months	Progressive loss of gait function. Concentration problems. Slight urine incontinence. 10 kg. weight loss (in 6 months).	Light distal tetra paresis. Hyperreflexia bilateral. Slow gait with reduced step length and -height. Moderate dyscalculia. MMSE and MDRS according to age. Tap test (30 ml.) with no effect. No LIT.	MRI = Dilated lateral and 3rd ventricles without cortical atrophy. Periventricular changes. White matter gliosis in basal ganglions, pons and mesencephalon without post contrast enhancement.	Negative.	Positive.	15.2	130 (Lymphocytic overweight).	380	SPECT = only a weak perfusion deficiency left frontal lope compared to the right. Not significant
2003 [14]	Male	76	6 months.	Progressive cognitive decline, weight loss and increasing falls because of imbalance. Debut of urine incontinence.	Bilateral ataxia. Amnesia for recent events. Spatiotemporal disorientation. MMSE =15/30 MDRS = 98/144 Tap test (50 ml.) with no effect. No LIT.	MRI = Dilated ventricles. Suggesting NPH.	Not reported	Positive.	19.7	250 (60% lymphocytes).	3000	Direct examination and cultures for usual bacteria in the blood and CSF = negative. PCR of CSF = negative for CVM, VZV, Epstein Barr, and herpes simplex viruses. Serological tests for syphilis = negative.
2004 [18]	Female	83	6 months	Weight loss of 5–7 kg. Urine incontinence. Gait instability. Slight diplopia.	Impaired memory and word finding tested via CERAD. MMSE = 18/30, Tap test (unknown ml.) with effect. No LIT.	MRI = Enlarged ventricles suspicious for NPH.	Not reported	Positive	Significantly elevated	69	3542	Not described.
2008 [15]	Female	80	6 months.	Progressive loss of memory and gait problems, now needing support to walk. Normal bladder control.	Slow, wide based gait with short shuffling steps. Turning nearly impossible. Bilateral mild ataxia. Reduces attention. Amnesia for recent events Spatiotemporal disorientation. MMSE =21/30. Tap test (50 ml.) = markedly effect. No LIT.	MRI = Dilated ventricles and periventricular lesions No post contrast enhancement. Suggesting NPH.	Positive.	Positive.	Significantly elevated.	45 (90% lymphocytes).	Elevated.	Not described.
2011 [19]	Female	71	9 months	Progressive confusion and lability of mood. Memory loss to dependency of daily living. Weight loss of 15 kg, and daily nausea. Gait instability with falls. Debut of urine incontinence (2 weeks).	MMSE = 17/30, IDSR = 18 (if 7–22, suspicion for Alzheimer’s dementia), Loss of second language. Tap test or LIT not reported.	MRI = atrophic. Expansion of the ventricles. Cella media index = 3,4 (abnormal if < 4). Bilateral symmetric mesial temporal lobe atrophy.	Positive	Positive	7.0	964 (lymphocytic overwight)	2351	SGDS = 8 (mild depression). No effect of 4 months antidepressants. FDG-PET-C. = normal for age. Pupillooccilation = no sign of dementia. Gastroscopy and coloscopy i.a. ANA, HCV-Ab., HIV-Ab, Syphillis Ab = negative
2016 [16]	Female	75	10 months.	Abdominal discomfort, nausea, cognitive decline, occasional urine incontinence.	Rigor and bradykinesia in the right arm. Unsteady, broad-based, short-stepped gait with forward flexed trunk posture. Mildly painful nuchal rigidity, Low mood. Not fully oriented to time. Slowing of speech and movements with word-finding difficulties. MMSE = 20/30 Tap test or LIT not reported.	MRI = mild periventricular white matter changes and slight widening of the lateral ventricles compared to cerebral sulci with a borderline Evans’ index of 0.34 (normal< 0.3) indicating possible early NPH.	Not reported.	Positive.	Highly positive	Lymphocytic pleocytosis	Elevated.	Not described.
2018 [17]	Male	87	Not reported.	Urine incontinence and progressive weakness and gait problems.	Bilateral hand tremor, dysdiadokokinesis, dysmetria, not orientated in time and place. Tap test or LIT not reported.	CT = increase in the size of the third and lateral ventricles suggesting communicating hydrocephalus superimposed on cerebral atrophy secondary to chronic lacunar infarcts. MRI = ventriculomegaly was more likely due to cerebral atrophy than balanced hydrocephalus.	Positive.	Negative.	Not reported.	Lymphocytic pleocytosis	Not reported.	Not described.
Our study (case from 2018)	Male	67	6 months.	Progressive balance problems, slight cognitive complaints, muscle soreness, urine urge.	Imbalance when walking on a line. Left sided ataxia OE and UE. Hyperreflexia. Word latency. MMSE = 28/30, ACE = 89/100 (affected animal fluency and s-word fluency). Tap test not performed, but improvement in some symptoms after first LP. No LIT.	MRI = dilated lateral and third ventricles, periventricular hyperintensity but no other pathology, especially no post contrast enhancements. EI = 0,377, CA = 117^o^	Negative.	Positive.	11.7	118 (80% Lymphocytes).	93	CSF cultured for bacteria and fungus = negative. Tests for both HIV (PCR and DNA), cryptococcus (antibodies), HSV and VZV (PCR and antibodies) and mycobacteria and Tuberculosis (PCR) = negative. Flowcytometry = sign of reactive response, but not a malignant immune response. Serology for Syphilis = negative. S-ACE for Sarcoidosis = negative

^a^A positive Tap-test is a removal of CSF by LBP, that gives an effect of the cognition or gait 30 min to 4 h afterward. *MMSE* Mini Mental Status Examination, *MDRS* Mattis Dementia Rating Scale, *CERAS* “ Consortium to Establish a Registry for Alzheimer’s ”, which is a cognitive test. The cella media index (Evans’ index) = ratio of biparietal diameter of skull to maximum external diameter of lateral ventricles at cella media, *SPECT* Single-Photon emission computed tomografhy, *PCR* Polymerase chain reaction studies, *CMV* Cytomegalo virus, *VZV* Varicella zoster virus, *S-ACE* Serum angiotensin converting enzyme, *LIT* Lumbar infusion test

As the early phase of NPH can present itself with only cognitive symptoms, and as NPH can be mistaken for the radiology of an atrophic, neurodegenerative brain [20], one could suspect that some of the cases of “Lyme induced dementia” [21, 22] or “neuropsychiatric symptoms of neuroborreliosis” [23] could in fact be patients having Bb induced sNPH. For these conditions the literature describes similar chronic meningitis with inflammation and infiltration as the cause and mechanism of symptoms [5, 24] and this could argument for some degree of diagnostic overlap or misdiagnosis. Furthermore, NPH is generally considered underdiagnosed.

The international guidelines of iNPH in 2005 [25] and the Japanese guidelines from 2004 [26] both have the Evan’s Index (EI) as an obligational criterion for the enlarged ventricular system, supporting the clinical criteria of NPH [27]. EI is the ratio of the transverse diameter of the anterior horns of the lateral ventricles to the greatest internal diameter of the skull and has a cut-off of ≥0.30.

Imagine features of the callosal angle (CA) of 90° or less at the level of the posterior commissure [25, 26], presence of periventricular hyperintensity and deep white matter intensity (DWMH), low flow void of 3rd or 4th ventricle, z-Evan’s Index (z-EI) ≥0,3, enlarged sylvian fissure and the narrowing of sulci and subarachnoid spaces over the high convexity (DESH) are all features that can increase the probability of iNPH [27].

The CSF dynamic test of (spinal) tap test and lumbar infusion test (LIT) has been used to both increase the diagnostic probability of iNPH but also to select the patient group that will profit from a potential shunt operation [26].

While the same diagnostic approach has been used for sNPH for many years, studies from 2017 [7, 8] show that not only does sNPH affect a younger patient group with faster progression of symptoms, but the ventricular system also expands in a symmetrical and proportional way with a lower total CSF-volume than iNPH and with diminished subarachnoid areas than the healthy controls. This concludes, that the features of CA, periventricular hyperintensity, DWMH, z-EI and DESH are less likely to develop and be present in sNPH than in iNPH, and as such suggested to be accounted less for in the diagnostics [7].

sNPH as a group is as well as iNPH being treated with neurosurgical procedure of shunt implantation and in fact with a better prognosis (75%) for symptom improvement [5].

Table 1 list the characteristics and diagnostics of each published case based on a review of the literature to date (see Fig. 2).

Only one of the prior cases reported a tick bite within a year before symptom debut [13], and the symptom duration at admission time ranged from approximately 4 months to 1 year.

All had intrathecal synthesis of Bb antibodies either IgG or IgM with lymphocytic CSF-pleocytosis and elevated CSF-protein (though the latter was not stated in one case [17]) and so fulfilled the consensus criteria for neuroborreliosis [28, 29].

All cases reported some degree of progressive gait and cognitive problems, and all but one [15] presented degrees of urinary incontinence.

Objectively all cases had disorientation or word latency to different degrees with the addition of either broad based gait or inability to walk in a line. Five out of 9 had mild to moderate ataxia, one had bradykinesia and rigor, and one had emotional lability and one presented with abnormal almost psychotic behaviour. Of diagnostics all had dilated lateral ventricles, but not all articles specified the radiology in detail. In one case only was the EI noted. Two cases (including ours) reported a normal opening pressure at LBP, while the measurement was not reported in the rest.

Neither of the prior published cases nor the case presented in this article were treated with ventricular shunt, probably because of the clear effect of the AB. All except our case was treated with intravenous ceftriaxone 2 g./day for between 2 and 5 weeks (the general agreement of treatment of Lyme disease [1]). All were eventually described with either full recovery or very limited non-invalidating remaining symptoms.

In only two cases did the radiological findings (12 and 18 months after AB) follow the clinical improvement with decreased dilatation of the lateral ventricles. In 4 cases, the control scan was unchanged, while 3 cases didn’t make a control scan.

In conclusion, this article is a rare presentation of chronic neuroborreliosis in the shape of sNPH, that have similar clinical presentation and treatment outcome as the prior 6 published cases. It shows the importance of early diagnosis and distinction from iNPH, as the cheap and minimally invasive procedure of LBP can shorten the symptom duration and completely prevent an unnecessary surgical intervention.

## Data Availability

Raw data were generated at the patient registry of the public hospitals in Denmark. Derived data supporting the findings of this study are available from the corresponding author LNG on request.

## Abbreviations

AB: Antibiotic
ACE: Addenbrooke’s Cognitive Examination
Bb: Borrelia Borgdorferi
CA: Callosal angle
CRP: C reactive protein
CSF: Cerebrospinal fluid
DESH: Disproportionately enlarged subarachnoidal space
DNA: Deoxyribonucleic acid
DWMH: Deep white matter intensity
EI: Evans Index
GP: General Practitioner
HIV: Human immunodeficiency virus
HSV: Herplex simplex virus
IgG, IgM: Immunoglobulin type G, immunoglobulin type M
iNPH: Idiopathic normal pressure hydrocephalus
LBP: Lumbar puncture
LIT: Lumbar infusion test
MMSE: Minimal Mental State Examination
MRI: Magnetic resonance imaging
NPH: Normal pressure hydrocephalus
PCR: Polymerase chain reaction
R-OUT: Outflow resistance
S-ACE: Serum angiotensin converting enzyme
sNPH: Secondary normal pressure hydrocephalus
VZV: Varicella zoster virus
